# Thioanisole ester based logic gate cascade to control ROS-triggered micellar degradation†

**DOI:** 10.1039/d2py00207h

**Published:** 2022-03-30

**Authors:** Irene Piergentili, Pepijn R. Bouwmans, Luuk Reinalda, Reece W. Lewis, Benjamin Klemm, Huanhuan Liu, Robin M. de Kruijff, Antonia G. Denkova, Rienk Eelkema

**Affiliations:** Delft University of Technology, Department of Chemical Engineering Van der Maasweg 9 2629 HZ Delft The Netherlands r.eelkema@tudelft.nl; Delft University of Technology, Department of Radiation Science and Technology Mekelweg 15 2629 JB Delft The Netherlands

## Abstract

In certain tumor and diseased tissues, reactive oxygen species (ROS), such as H_2_O_2_, are produced in higher concentrations than in healthy cells. Drug delivery and release systems that respond selectively to the presence of ROS, while maintaining their stability in “healthy” biological conditions, have great potential as on-site therapeutics. This study presents polymer micelles with 4-(methylthio)phenyl ester functionalities as a ROS-responsive reactivity switch. Oxidation of the thioether moieties triggers ester hydrolysis, exposing a hydrophylic carboxylate and leading to micellar disassembly. At 37 °C, the micelles fall apart on a timescale of days in the presence of 2 mM H_2_O_2_ and within hours at higher concentrations of H_2_O_2_ (60–600 mM). In the same time frame, the nanocarriers show no hydrolysis in oxidant-free physiological or mildly acidic conditions. This logic gate cascade behavior represents a step forward to realize drug delivery materials capable of selective response to a biomarker input.

## Abstract 2

Thioanisole ester polymer side chains hydrolyze exclusively upon thioether oxidation, showing logic gate response. ROS-induced ester hydrolysis on the hydrophobic domain leads to nanocarrier disassembly with potential for targeted drug release.

## Graphical abstract

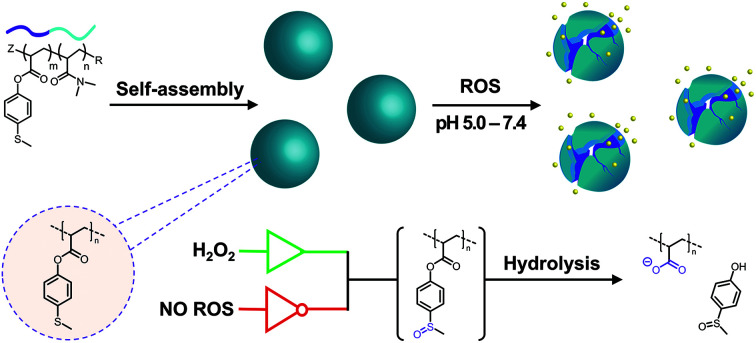

## Introduction

Smart materials that respond to external stimuli have emerged as an efficient platform to obtain targeted nanotherapeutics. Historically, amphiphilic block copolymers that spontaneously self-assemble in an aqueous environment are used as carriers to solubilize important, but poorly water soluble anti-inflammation and anti-cancer drugs in the bloodstream.^1^ Still, these systems can suffer from nonspecific biodistribution and uncontrolled drug release, causing ineffective treatment or undesired side effects in the patient.^2^ Therefore, the need for personalized therapeutics inspired researchers to study materials responsive to abnormal biological changes specifically caused by the diseased cells. Over the last decades, intelligent polymers have been developed to be responsive to several stimuli, like pH,^3^ temperature,^4^ and small molecule or biomacromolecular signals.^5^

Reactive oxygen species (ROS), such as hydrogen peroxide, regulate fundamental physiological processes in cells, including oxygen metabolism and signaling pathways.^6,7^ However, in cancers and inflammatory,^6–8^ cardiovascular^9^ or neurodegenerative diseases,^10,11^ ROS are produced at a rate that natural antioxidant mechanisms, like enzymes (*e.g.* superoxide dismutase, catalase), cannot overcome.^12–14^ Elevated intracellular H_2_O_2_ concentrations in diseased tissues are typically between 10 and 100 μM,^15,16^ and can go up to 10 mM.^17^ This change in the oxidative state of the cellular environment can be used as a trigger for selective local cargo release.^18–22^ The pioneering work of Hubbell *et al.* in 2004 reported the first oxidation-sensitive polymeric vesicles for drug delivery purposes, degradable in 10 h in presence of 10 vol% H_2_O_2_.^23^ Since then, the same group have applied that principle in several organic nanoparticles, including micelles and vesicles.^24–26^ Their responsiveness is based on the oxidation of hydrophobic thioethers to more hydrophilic sulfoxides and sulfones. Oxidation leads to more water-soluble polymeric materials, and therefore, less stable micelles, allowing for the release of the incorporated cargo.^27^ In most of these examples, exceedingly high concentrations of H_2_O_2_ (2.0–10 vol%) are required to disassemble the carrier within hours.

In contrast, boronate-based polymers have been extensively studied in the last 10 years because their sensitivity to H_2_O_2_ is in the sub-millimolar range.^28,29^ Implementing boronic esters in a phenol-based polymeric backbone, Almutairi *et al.* reported in 2012 a cascade degradable nanoparticle sensitive to only 50 μM of H_2_O_2_.^30^ This unique example of a nanocarrier sensitive to biologically relevant concentrations of H_2_O_2_ was, however, accompanied by poor control over cargo release (non-specific release and no significant effect over the release time scale when different concentrations of H_2_O_2_ are used). Various mechanisms of H_2_O_2_ triggered drug release based on the boronate cleavage methodology have been developed, including the degradation of polymeric backbones,^31^ activation of prodrugs^32,33^ and destruction of the amphiphilic block copolymer structure, usually by unmasking a more hydrophilic aliphatic acid (*e.g.* polyacrylic acid).^34–36^ In addition, boronic esters are also susceptible to hydrolysis and glycolysis at mildly acidic pH, forming diols and boronic acids.^37^ The multi-responsiveness of boronates makes it a versatile moiety for biomedical materials, but can also pose a problem in terms of selectivity, causing off-target release. The need in the field of ROS-responsive materials resides currently in the design of systems with a cascade logic gate behavior, able to ensure specific and robust control over the performance of drug carriers.^38^

In this work, we present an oxidation-sensitive bond cleavage method that merges the responsivity of thioethers toward oxidation^39^ with the tunability of ester hydrolysis through a reactivity switch. In the design of the system, we chose thioanisole type groups as our ROS-responsive moieties. First, we considered that the oxidation potential of aromatic thioethers is in the ideal range to undergo oxidation by H_2_O_2_.^20,40^ The oxidation of aliphatic thioethers to sulfoxides or sulfones has been extensively applied in polymeric materials to increase the hydrophilicity of the chain.^23,41–44^ An aromatic ring adjacent to the thioether group could enhance the nucleophilicity of the sulfur atom towards H_2_O_2_.^45^ However, it is known that the oxidation of aromatic thioethers to the corresponding sulfoxide and sulfone is insufficient to achieve a desired solubility switch.^46^ Instead, we decided to use sulfide oxidation to increase the hydrolytic lability of a nearby ester, thereby introducing a more effective solubility switch. Knowing that electron withdrawing groups on the aromatic ring of phenyl acetate esters increase the electrophilicity of the ester, our idea was to achieve a reactivity switch when the electron donating thioether is oxidized into a more electron withdrawing group, such as the corresponding sulfoxide or sulfone.^47–50^ Therefore, H_2_O_2_-triggered thioether oxidation would activate the adjacent ester functionality towards hydrolysis.

**Fig. 1:**
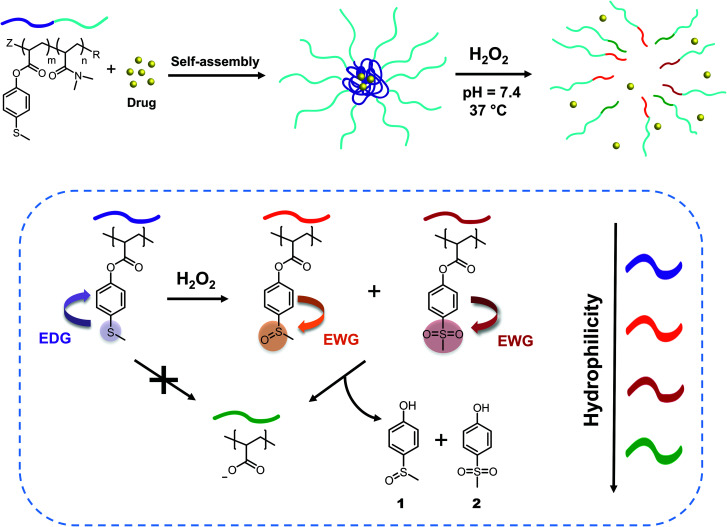
General concept: drug release from ROS-responsive micelles, triggered by the hydrolytic cleavage of ester bonds through switching from an electron donating (EDG) thioether group to electron withdrawing (EWG) sulfoxide and sulfone groups upon oxidation by H_2_O_2_.

We synthesized two amphiphilic block copolymers with different lengths of *N*,*N*-dimethylacrylamide as a hydrophilic block and 4-(methylthio) phenyl acrylates as a hydrophobic part of the chain. In aqueous environment, these macromolecules self-assemble into micelles with diameters between 30 and 50 nm, which is an appropriate size range for drug nanocarriers.^51^ When H_2_O_2_ is added, the oxidation of sulfide to sulfoxide leads to the removal of 4-(methylsulfinyl)phenol 1 and 4-(methylsulfonyl)phenol 2 units through hydrolysis, turning the hydrophobic core into a more hydrophilic acrylate anion block and finally obtaining micellar disintegration (Fig. 1). These ester-based polymeric micelles show great stability towards hydrolysis at neutral and acidic pH, demonstrating specific responsiveness towards oxidation by cascade logic behavior.

## Results and discussion

### Synthesis and characterization of p(DMA_*n*_-*b*-MTPA_*m*_)

**Scheme 1:**
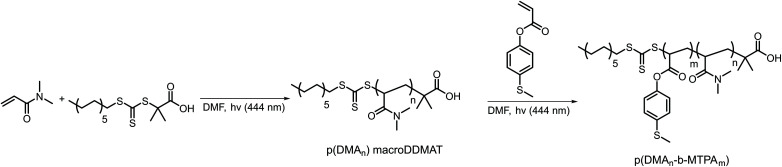
Synthetic route for preparation of ROS-responsive p(DMA_*n*_-*b*-MTPA_*m*_) diblock copolymers *via* light initiated RAFT polymerization.

We synthesized the amphiphilic p(DMA_*n*_-*b*-MTPA_*m*_) block copolymers through sequential light-initiated RAFT polymerization (Scheme 1).^52^ The choice of extending poly(*N*,*N*-dimethylacrylamide) macroDDMAT with 4-(methylthio) phenyl acrylate (MTPA) was due to the less successful chain extension when we attempted the opposite order. First, p(DMA) macroDDMAT was prepared using 2-(dodecylthiocarbonothioylthio)-2-methylpropionic acid (DDMAT) as the RAFT agent to obtain 130 and 102 DMA unit long polymeric chains (Table S1†). Then, the chain extension of hydrophilic macromolecular chain transfer agents p(DMA_130_) macroDDMAT and p(DMA_102_) macroDDMAT with MTPA (Table S2†) produced PM16 and PM32. ^1^H NMR spectra of the block copolymers in CDCl_3_ (Fig. S1†) showed characteristic (broadened) signals of both DMA and MTPA, with the ratio of their integrations in line with what was expected from conversion data. In agreement with the ^1^H NMR results, GPC traces (Fig. S2†) confirmed successful chain extension for both polymers through increase in molecular weight of a single peak. Thus, we obtained two block copolymers (Table 1), allowing investigation into the influence of varying hydrophobic block/hydrophilic block ratios on micelle formation and drug loading efficiency.^53^

**Table 1:** Characterization of the block copolymers p(DMA_*n*_-*b*-MTPA_*m*_)

Code	Polymer	*M*_n, conv_ (kDa)	*M*_n, GPC_ (kDa)	*Đ* (*M*_w_/*M*_n_)
DMA130	p(DMA_130_)	13.2	13.0	1.13
DMA102	p(DMA_102_)	10.5	11.1	1.28
PM16	p(DMA_130_-*b*-MTPA_16_)	16.3	16.0	1.16
PM32	p(DMA_102_-*b*-MTPA_32_)	16.7	17.4	1.27

### Preparation and characterization of p(DMA_*n*_-*b*-MTPA_*m*_) micelles

PM16 and PM32 micelles with a p(MTPA) core and a p(DMA) corona were prepared by a solvent evaporation method using THF. Addition of sodium phosphate buffer (PB, 100 mM, pH = 7.4) to the solubilized polymers led to micellar dispersions of PM16 and PM32. The average hydrodynamic diameter (*D*_H_) of the micelles at 1.0 mg mL^−1^ measured by DLS was 31.6 ± 0.5 and 42.4 ± 0.9 nm for PM16 and PM32, respectively (Table 2). With PM32 showing a larger *D*_H_, the hydrodynamic size appeared to be correlate with the length of the hydrophobic block.^54^ TEM images (Fig. 3B and D) acquired from micellar dispersions at 1.0 mg mL^−1^ demonstrated the formation of spherical particles, ascribable to micelles. The particle analysis based on these TEM images gave an average diameter of 17.7 ± 3.1 nm for PM16 (Fig. S5A†) and 25.8 ± 3.1 nm for PM32 (Fig. S5B†). Cryo-EM analysis further confirmed the spherical morphology of both PM16 (Fig. S6B†) and PM32 (Fig. S7C†) micelles, with an average diameter of 10.4 ± 1.2 (Fig. S6A†) and 19.2 ± 2.3 nm (Fig. S7A†), respectively. Combined, these analyses demonstrated that both polymers formed micelles with the appropriate size range for nanotherapeutics,^55^ and are thus possibly loadable with hydrophobic cargo.^56^

**Table 2:** Size of PM16 and PM32 micelles measured by DLS, TEM and Cryo-EM

Polymer	*D*_H_ (nm)	*D*_TEM_ (nm)	*D*_Cryo-EM_ (nm)
PM16	31.6 ± 0.5	17.7 ± 3.1	10.4 ± 1.2
PM32	42.4 ± 0.9	25.8 ± 3.1	19.2 ± 2.3

### H_2_O_2_ induced oxidation and hydrolysis of p(DMA_*n*_-*b*-MTPA_*m*_) micelles

**Fig. 2:**
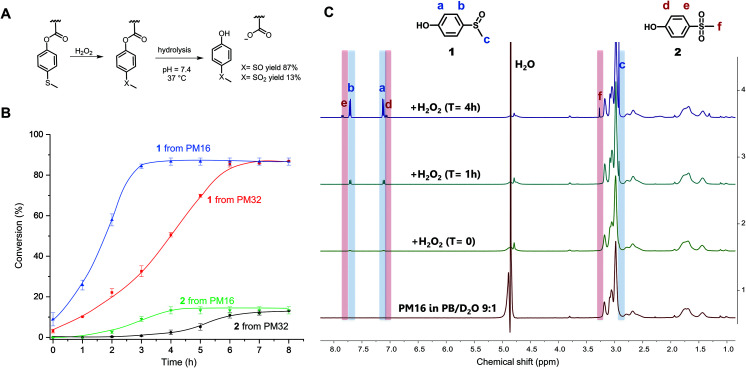
(A) Scheme of H_2_O_2_-triggered solubility switch of 4-(methylthio) phenyl acrylate by oxidation induced hydrolysis leading to formation of hydrophilic acrylate anion and removal of 1 and 2 from the polymers. (B) Conversion measured through ^1^H NMR spectroscopy of 1 and 2 upon the addition of 2.0 wt% of H_2_O_2_ to PM16 and PM32 micellar solutions (6.8 mg mL^−1^) in PB (100 mM, pH = 7.4)/D_2_O 9 : 1 at 37 °C. The curves are drawn as a guide for the eye. (C) ^1^H NMR in PRESAT configuration of PM16 micelles after treatment with 2.0 wt% of H_2_O_2_ in PB (100 mM, pH = 7.4)/D_2_O 9 : 1 at 37 °C.

After characterization of the micelles, we wanted to test their response to H_2_O_2_. The oxidation of organic thioethers with H_2_O_2_ is notably slow and depends on the concentration of both reactants.^26,57^ Thus, we chose to use a large excess of H_2_O_2_ (90 equivalents for PM16 and 46 for PM32) compared to the thioether units of the polymers to obtain an overview of the response times and behavior of these micelles. PM16 (6.7 mM thioether units at 6.8 mg mL^−1^) and PM32 micelles (13 mM thioether units at 6.8 mg mL^−1^) in PB/D_2_O 9 : 1 were combined with 2.0 wt% H_2_O_2_ (600 mM) at 37 °C and studied by ^1^H NMR spectroscopy (Fig. 2C and Fig. S8† for PM16 and PM32, respectively). The micelles in aqueous media (bottom spectrum, Fig. 2C) showed only the p(DMA_*n*_) peaks, caused by the core–corona structure that is typical of polymeric micelles. However, almost immediately after the addition of H_2_O_2_, the ^1^H NMR spectra revealed the release of 1 (^1^H NMR spectrum reference in ESI†), confirming the oxidation and hydrolysis of the 4-(methylthio)phenyl ester functionalized core of the micelles (Fig. 2A).

Fig. 2B shows the results of the combined ^1^H NMR experiments for both polymeric micelles to give a comparative overview of the kinetics for different hydrophobic/hydrophilic block ratios. PM16 micelles exhibited 100% of degradation of the 4-(methylthio)phenyl ester moieties 3 h after addition of H_2_O_2_, converting to 87% of 1 and 13% of 2. PM32 micelles reached the same outcome after 6 h. It is worth noting that the release of these hydrolysis products followed sigmoidal curves. This effect was more significant for the release of 2, which showed lag times of 2 h for PM16 and 4 h for PM32.

The conversion to 9% of 1 from PM16 and 3% from PM32 in the first ^1^H NMR acquisition after the addition of H_2_O_2_ (∼5 minutes) would suggest that as soon as the oxidation of the sulfide groups occurred, hydrolysis took place as well. This hypothesis is also supported by the absence of broad peaks related to the poly sulfoxide/sulfone in all the spectra acquired. In addition, both polymeric micelles resulted in the same distribution of sulfoxide and sulfone at the end of the degradation, which could be an indication that PM16 and PM32 followed a similar oxidation/hydrolysis mechanism. To asses this hypothesis, it is also interesting to note that PM32 required almost exactly 2 times as long to hydrolyze as PM16, matching the corresponding number of the 4-(methylthio)phenyl ester units to oxidize. At the same time, no clear dependence on hydrophilic block length was observed. The formation of degradation products follows sigmoidal curves, which is in line with the fact that both oxidation of thioethers and ester hydrolysis inside polymer micelles in aqueous environment are known to be autocatalytic processes.^25,58^ At the macromolecular level, when the hydrophobic core becomes more hydrophilic due to sulfur oxidation and ester hydrolysis, the micellar core turns into a more soluble matrix for H_2_O_2_. Thus, the increase of the local concentration of oxidant causes the acceleration of the reaction rate.^25,59^ A clear indication of this phenomenon was the acceleration that we observed for the approximate complete release of 2 from PM32 in 2 h, after a 4 h long lag time. In fact, the significantly longer lag time for PM32 than PM16 can easily be explained considering the larger and less accessible hydrophobic core.

### Stability of p(DMA_*n*_-*b*-MTPA_*m*_) micelles

To assess the stability of the micelles in non-oxidative physiological conditions (pH 7.4, 37 °C), we followed the hydrolysis rate for both PM16 and PM32 micelles by ^1^H NMR for 144 h (6 days). We did observe the formation of a small amount of 1, 1.0% for PM16 micelles and 0.7% for PM32 micelles after 6 days (Table 3). Furthermore, we investigated the hydrolytic stability of the ester functions in PM16 and in PM32 at pH 5.0 and 6.0 (37 °C), to analyze their behavior in acidic environments, which may occur in tissues or cells. Encouragingly, in all conditions both micelles were found to be hydrolytically stable, with ≤1.3% of 1 released in all cases after 6 days (Table 3). For all the experiments, the absence of the characteristic peaks of 4-(methylthio)phenol (reference spectrum in ESI†) showed that the 4-(methylthio)phenyl ester units do not directly hydrolyze. On the other hand, the release of 1 indicated background oxidation of the sulfide groups attached to the polymeric chain, enabling hydrolysis of the esters. This would demonstrate that the hydrolysis occurs exclusively after the oxidation of the thioether moiety. Nevertheless, such phenomenon can be considered negligible compared to the effect of the addition of H_2_O_2_ reported above, in which the hydrolysis of the pendent esters was complete within hours. Overall, we could confirm that PM16 and PM32 micelles are resistant to direct hydrolysis of 4-(methylthio)phenyl esters in environments with pH ranging from 5.0 to 7.4, demonstrating a unique response to oxidative stimulus.

**Table 3:** Oxidant-free release (%) of 1 from PM16 and PM32 micelles after 6 days at different pH

pH	Release of 1 from PM16 (%)	Release of 1 from PM32 (%)
7.4	1.0	0.7
6.0	1.3	0.9
5.0	1.1	0.8

### Morphological study of oxidation of p(DMA_*n*_-*b*-MTPA_*m*_) micelles

**Fig. 3:**
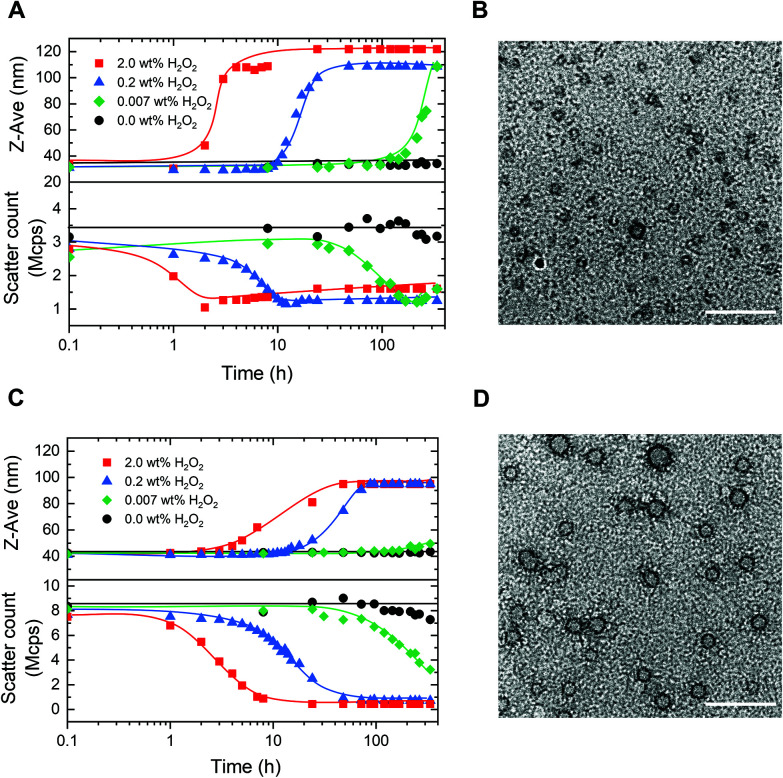
Morphological study of oxidation of PM16 and PM32 micelles. The curves are drawn as a guide for the eye. (A) *Z*-Average diameter (top) and scatter count (bottom) of PM16 micelles (0.9 mg mL^−1^) measured by DLS at 37 °C for four concentrations of H_2_O_2_: 2.0 wt% (■ red line), 0.2 wt% (▲ blue line), 0.007 wt% (◆ green line) and 0.0 wt% (control ● black line). (B) TEM image (scale bar = 100 nm) of PM16 micelles at *t* = 0, stained with 2.0 wt% uranyl acetate. (C) *Z*-Average diameter (top) and scatter count (bottom) of PM32 micelles (0.9 mg mL^−1^) measured by DLS at 37 °C for four concentrations of H_2_O_2_: 2.0 wt% (■ red line), 0.2 wt% (▲ blue line), 0.007 wt% (◆ green line) and 0.0 wt% (control ● black line). (D) TEM image (scale bar = 100 nm) of PM32 micelles at *t* = 0, stained with 2.0 wt% uranyl acetate.

Having established the concept, we studied the morphological response of the micelles to various concentrations of H_2_O_2_. PM16 micelles (0.9 mM thioether units at 0.9 mg mL^−1^) were exposed to concentrations of 2.0, 0.2 and 0.007 wt% of H_2_O_2_ (DLS, Fig. 3A), corresponding respectively to 600, 60 and 2 mM. Upon addition of 2.0 wt% H_2_O_2_, we could not observe changes in *Z*-average diameter in the first hour (Fig. 3A top, ■ red line). However, the scatter count dropped from 2.8 to 2.0 Mcps (Fig. 3A bottom, ■ red line), indicating that the micelles started to dissociate. This value dropped to 1.0 Mcps within the next hour. After 4 h, the PM16 micelles reached a maximum *Z*-average diameter of 108 nm. The approximate 3-fold reduction in scatter count observed after H_2_O_2_ addition indicates degradation of the micelles due by oxidation induced hydrolysis. While the concurrent increase in *Z*-average diameter may be counterintuitive, it can be explained by a partial clustering of the hydrolyzed polymer chains. TEM images showed the presence of micelles before H_2_O_2_ addition (Fig. 3B), and no significant structure could be detected 24 h after the addition of 2.0 wt% H_2_O_2_ (Fig. S5C†). This analysis supported the rapid disruption (within 4 hours) of the polymeric micelles after addition of 2.0 wt% H_2_O_2_, as showed in both DLS and ^1^H NMR data. *Z*-Average diameter and scatter count after 24 h (Fig. 3A, ▲ blue line) of PM16 micelles triggered with 0.2 wt% H_2_O_2_ were similar to those observed for 2.0 wt% in the first 4 hours. Interestingly, this could be interpreted as ∼6 fold reduction in rate of disassembly of the micelles when the concentration of H_2_O_2_ is 10 times lower.

Encouraged by these promising results, we decided to investigate whether the system is able to respond to concentrations of the oxidant approaching biologically relevant conditions (0.007 wt% (2 mM) of H_2_O_2_). *Z*-Average diameter of PM16 micelles reached a maximum after 336 h (Fig. 3A top, ◆ green line) and the scatter count decreased steadily from 48 h, getting to 1.2 Mcps at 168 h (Fig. 3A bottom, ◆ green line). It is important to note that, in the absence of H_2_O_2_ the PM16 micelles remained stable at 32–34 nm and 3.2–3.7 Mcps for 336 h. These results demonstrate a sensitivity down to 0.007 wt% H_2_O_2_ and a considerable stability in the absence of an oxidative trigger.

Next, we repeated the same DLS study with PM32 micelles (1.8 mM thioether units at 0.9 mg mL^−1^). These micelles had similar behavior to PM16 micelles, with a sigmoidal increase in *Z*-average diameter and a sigmoidal decrease in scatter count after H_2_O_2_ addition. Specifically, the *Z*-average diameter of PM32 micelles increased from 42 to ∼90 nm (Fig. 3C top), while the scatter count dropped from 8.0 to 1.0 Mcps (Fig. 3C bottom). The lower plateau value of the scatter count was reached 7 and 48 h after the addition of 2.0 wt% H_2_O_2_ and 0.2 wt% H_2_O_2_, respectively. Showing that, similarly to PM16, PM32 micelles disrupted ∼6 times slower when 10 times lower oxidant concentration was used. For 0.007 wt% (2.0 mM) H_2_O_2_, the scatter count dropped to 3.2 Mcps after 336 h (Fig. 3C bottom, ◆ green line). Considering that here the ratio of thioether units/H_2_O_2_ was nearly 1, the low rate of micellar disruption is not surprising. Additionally, like for PM16 micelles, the scatter count remained relatively stable (7.3–8.5 Mcps) over 336 h without H_2_O_2_.

Curiously, the aggregates formed after the disruption of PM32 were apparently smaller than those obtained from PM16. With TEM images of PM32 micelles before (Fig. 3D) and 24 h after the addition of 2.0 wt% H_2_O_2_ (Fig. S5D†), we could see the initial spherical micelles, but could not distinguish any particular structure after the H_2_O_2_ treatment. We therefore acquired Cryo-EM images of PM32 micelles before (Fig. S7C†) and 24 h after (Fig. S7D†) the addition of 0.2 wt% H_2_O_2_. The particle analysis showed a relatively small increase in average diameter from 19.2 ± 2.3 (Fig. S7A†) to 28.9 ± 15.4 nm (Fig. S7B†), supporting the increase in size during PM32 micelles degradation. On the other hand, Cryo-EM images (Fig. S6†) of PM16 micelles before and 24 h after the addition of 0.2 wt% H_2_O_2_ exhibited the starting spherical and homogeneous micelles in non-oxidative conditions, but, similar to the TEM images, did not show any significant structure after the H_2_O_2_ addition. The low scatter count associated with the proposed larger aggregates indicates a very low abundance, explaining the result of Cryo-EM imaging. Despite of the uncertain characterization of the final structures, the *Z*-average diameter change and the decrease in scatter count of the micelles at different concentrations of H_2_O_2_ measured by DLS demonstrated the oxidation-triggered morphological change of both PM16 and PM32 micelles.

Interestingly, the DLS data not only agreed with the ^1^H NMR results, but also followed similar sigmoidal trends, confirming the autocatalytic degradation of our micelles. We observed that micelles prepared from PM16 underwent a faster (2–4 times depending on the conditions and methods of measurements) disassembly than those from PM32. The greater H_2_O_2_ sensitivity observed for PM16 indicates that shorter 4-(methylthio)phenyl ester functionalized blocks allow for faster micellar degradation. In perspective, this opens the possibility of tuning the hydrophobic block length of p(DMA_*n*_-*b*-MTPA_*m*_) to precisely control drug release.

### Assessment of Nile Red loading and release

To assess the suitability of p(DMA_*n*_-*b*-MTPA_*m*_) micelles as carriers for drug release, we chose Nile Red, a non-water soluble dye which is fluorescent exclusively in a hydrophobic environment. The fluorescence of Nile Red can be constant in presence of up to 5 vol% (∼7.3 wt%) H_2_O_2_ over 170 h,^60^ making it a good drug model for the time range and conditions of our experiments. First, we determined the drug loading (DL) and encapsulation efficiency (EE) of Nile Red using a known fluorescence method.^60^ We obtained DL (2.0–4.0 μg mg^−1^ polymer) and EE (10–20%) (Table S3†), comparable to other drug release systems reported in literature.^27^

**Fig. 4:**
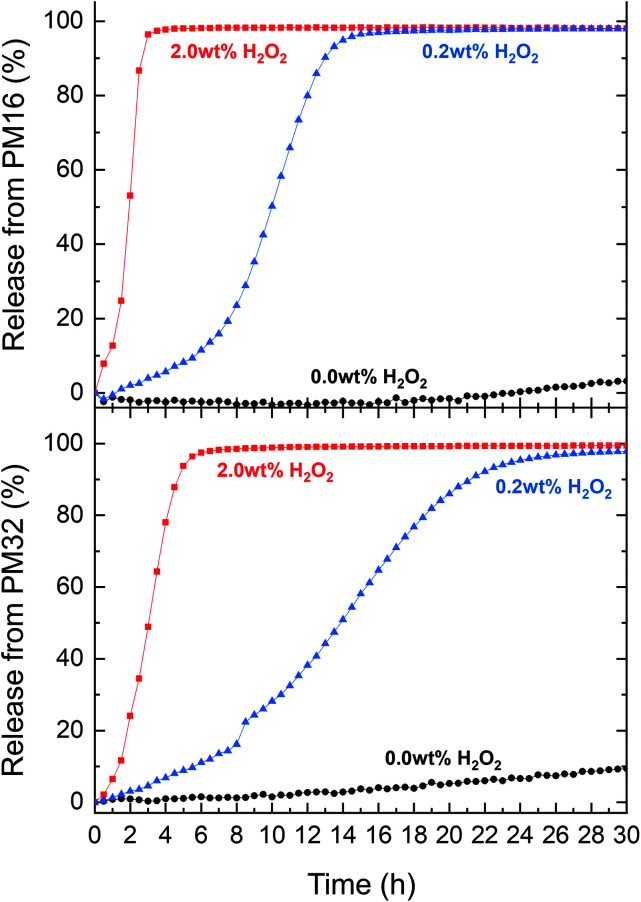
Nile Red release from PM16 (top) and PM32 (bottom) micellar dispersion (0.9 mg mL^−1^) in PB (100 mM, pH = 7.4) for three concentrations of H_2_O_2_ at 37 °C: 2.0 wt% (■ red line), 0.2 wt% (▲ blue line) and 0.0 wt% (control ● black line) measured by fluorescence spectroscopy (*λ*_ex_ = 540 ± 20 nm, *λ*_em_ = 620 ± 30 nm).

We subsequently tested the Nile Red loaded p(DMA_*n*_-*b*-MTPA_*m*_) micelles for release of Nile Red under oxidative conditions. PM16 micelles led to a 90% Nile Red release within 3 and 13 h when 2.0 wt% and 0.2 wt% of H_2_O_2_ was respectively added (Fig. 4, top). PM32 micelles released Nile Red on longer time scales (Fig. 4, bottom part), getting to 90% within 5 h (2.0 wt% H_2_O_2_) and 21 h (0.2 wt% H_2_O_2_). These results showed, similarly to the ^1^H NMR data, that PM16 micelles disassembled and released the cargo almost 2 times faster than PM32 micelles in presence of the same H_2_O_2_ concentration. We again would like to highlight that the Nile Red release curves presented sigmoidal shapes, in line with the data acquired with the previous techniques. Specifically the release profile from PM16 micelles at 0.2 wt% of H_2_O_2_ displayed a three stage profile, typical of polymeric drug delivery systems with a heterogeneous degradation mechanism.^61^ This would position the p(DMA_*n*_-*b*-MTPA_*m*_) micelles as an oxidation-triggered alternative to the hydrolysis-degradable polymers commonly used for controlled drug release.^62^ Interestingly, this behavior does not show any burst release,^63^ increasing the relevance of the system for applications where burst release of cytotoxic drugs may cause excessive side effects.^62^ We observed 10% release for PM32 micelles in a non-oxidative environment and 3% for PM16 by 30 h. This would suggest only minor passive leaking of Nile Red from the micelles. However, such effect can be considered negligible when compared to the release rate obtained in presence of H_2_O_2_. Overall, the release profile shows a dependence on the type of polymer and on the concentration of H_2_O_2_, making this technology potentially tunable according to the required dosage and release time of the drug.

### Cell viability assay of p(DMA_*n*_-*b*-MTPA_*m*_) micelles

We tested the cytotoxicity of PM16 and PM32 micelles on HeLa cells, by administering micellar dispersions in concentrations between 0.0 and 1.0 mg mL^−1^, in line with the concentration used for the morphological study and Nile Red release. After 24 h of incubation, the WST-8 assay showed high cell viability for both polymers across all the applied concentrations, with no statistical difference compared to the controls (Fig. S10†).

## Conclusion

In this work, we demonstrate selective ROS triggered breakdown of block copolymer micelles, and associated release of model cargo. The disruption mechanism is programmed into the material using a solubility switch in the hydrophobic block, based on a logic gate that increases the ester hydrolytic lability upon oxidation of a thioether phenyl moiety. In the absence of oxidants, such as H_2_O_2_, the micelles are stable for several days under neutral and mildly acidic buffered conditions (pH 5.0–7.4, 37 °C). Millimolar concentrations of H_2_O_2_ lead to micellar disintegration and cargo release on timescales of hours to days depending on the ROS concentration. Building on these encouraging results, our laboratory is currently investigating methods to further increase the ROS sensitivity of these micelles to concentrations typically present in cancerous tissue. Subsequently, studying the release of bioactive compounds and the stability of the polymers in blood plasma (specifically against esterase activity) will be necessary before *in vivo* evaluation. In perspective, the thioanisole ester-based logic gate could also be implemented as a linker in ROS-responsive prodrugs to achieve the release of the active compound, directly controlled by the oxidation-induced ester hydrolysis.

## Author contributions

Irene Piergentili: conceptualization, investigation, data curation, visualization, writing – original draft and writing – review & editing. Pepijn R. Bouwmans: investigation. Luuk Reinalda: investigation. Reece W. Lewis: supervision, conceptualization and writing – review & editing. Benjamin Klemm: data curation and visualization. Huanhuan Liu: investigation. Robin M. de Kruijff: investigation. Antonia G. Denkova: supervision, resources and writing – review & editing. Rienk Eelkema: supervision, conceptualization, resources and writing – review & editing.

## Conflicts of interest

The authors declare no conflicts of interest.

## Supplementary Material

PY-013-D2PY00207H-s001
