# Supplementary material for: Thioanisole ester based logic gate cascade to control ROS-triggered micellar degradation
Source: Polym Chem. 2022 Mar 30;13(16):2383–90. doi: 10.1039/d2py00207h (PMC9016795; doi:10.1039/d2py00207h)
Supplement: PY-013-D2PY00207H-s001 [file PY-013-D2PY00207H-s001.pdf]

Electronic Supplementary Material (ESI) for Polymer Chemistry.
This journal is © The Royal Society of Chemistry 2022

Supporting Information

Thioanisole Ester Based Logic Gate Cascade to Control ROS-triggered Micellar Degradation

Irene Piergentili[a], Pepijn R. Bouwmans[a], Luuk Reinalda[a], Reece W. Lewis[a], Benjamin Klemm[a], Huanhuan Liu[b], Robin M. de Kruijff[b], Antonia G. Denkova[b], Rienk Eelkema[a,*]

[a] Delft University of Technology, Department of Chemical Engineering, Van der Maasweg 9, 2629 HZ Delft, The Netherlands
[b] Delft University of Technology, Department of Radiation Science and Technology, Mekelweg 15, 2629 JB Delft, The Netherlands

Table of Contents

S1. Materials S3

S2. Synthesis S3

S2.1 Synthesis of 4-(methylsulfinyl)phenol[1] S3

S2.2 Synthesis of 4-(methylthio)phenylacrylate S3

S2.3 Synthesis of p(DMA_n) macro-DDMAT S4

S2.4 Synthesis of p(DMA_n-b-$MTPA_m$) S4

S3. Polymer Characterization S4

S3.1 ^{1}H NMR spectra of the block copolymers S5

S3.2 GPC data of the block copolymers S5

S4. Characterization of polymeric micelles S6

S4.1 Preparation of the polymeric micelles S6

S4.2 DLS measurements of the polymeric micelles before and after H_2O_2 treatment S6

S4.3 TEM images of the polymeric micelles before and after H_2O_2 treatment S7

S4.4 Cryo-EM images of the polymeric micelles before and after H_2O_2 treatment S8

S5. ^{1}H NMR study of p(DMA_n-b-$MTPA_m$) micelles S9

S5.1 ^{1}H NMR study of p(DMA_n-b-$MTPA_m$) micelles before and after H_2O_2 treatment S9

S5.2 ^{1}H NMR study of p(DMA_n-b-$MTPA_m$) micelles at different pH S10

S6. Drug load and release of p(DMA_n-b-$MTPA_m$) micelles S10

S6.1 Determination of micelle loading with Nile Red S10

S6.2 H_2O_2-triggered release of Nile Red from p(DMA_n-b-$MTPA_m$) micelles S11

S7. Cell viability assay on p(DMA_n-b-$MTPA_m$) micelles S11

S8.0 ^{1}H-NMR and ^{13}C-NMR spectrum for synthesized compounds S13

S1. Materials

All reagents were obtained from commercial suppliers (Sigma Aldrich, TCI Chemicals or Acros Organics) and used without further purification unless otherwise specified. Reference compounds 4-(methylthio)phenol and 4-(methylsulfonyl)phenol were purchased respectively from Sigma Aldrich and TCI. SDS of these compounds reports that chemical, physical, and toxicological properties have not been thoroughly investigated. 4-(Methylmercapto)phenol: this substance/mixture contains no components considered to be either persistent, bioaccumulative and toxic (PBT), or very persistent and very bioaccumulative (vPvB) at levels of 0.1% or higher. Air and moisture sensitive reagents were transferred via syringe. All air and/or moisture sensitive reactions were carried out in oven-dried glassware under a positive pressure of argon gas with commercially available anhydrous solvents. Petroleum ether refers to the fraction boiling in the range 40 – 60 °C. Reactions were monitored by analytical thin-layer chromatography (TLC) on silica gel plates (Merck $60F_{254}$) and either visualized by UV light (254 nm) or by staining with a solution of $KMnO_4/K_2CO_3$/AcOH in water followed by heating. Flash chromatography was performed on 230-400 mesh silica gel (Sigma Aldrich). ^1H NMR and ^{13}C NMR spectra were recorded on an Agilent-400 MR DD2 (400 MHz and 101 MHz for ^1H and ^{13}C, respectively) spectrometer at 298 K. Chemical shifts are reported in ppm relative to the residual solvent peak, the multiplicity is reported as follows: s = singlet, d = doublet, t = triplet, q = quartet, m = multiplet, and J-couplings (J) are reported in Hertz (Hz). To suppress the water peak, PRESAT configuration (suppress one highest peak) was used. NMR spectra were processed by MNova NMR software (Mestrelab Research). Infrared spectra were recorded on a FT-IR Thermo Fisher Nicolet 6700 spectrophotometer and are reported in wavenumbers. GC-MS samples were analyzed using an Agilent 5977 GC/MSD equipped with a Stabilwax MS column (oven temperature: 250 °C, flow: 2.5 mL/min). ESI-MS was performed using LTQ XL spectrometer equipped with Shimadzu HPLC setup operating at 0.2 mL/min flow rate with water/MeCN mobile phase containing 0.1 vol% formic acid and Discovery C18 column. Gel permeation chromatography (GPC) was performed on a Shimadzu system equipped with a LC-20AD liquid chromatograph and a RID-10A refractive index detector. Fluorescence release was measured in 96 well plates using a micro plate reader (Biotek Synergy H1). Fluorescence spectra of Nile Red loading were recorded with a fluorescence spectrometer Spex Fluorolog-3 equipped with a standard 90° setup. Dynamic light scattering (DLS) measurements were performed on a Malvern Zetasizer Nano-ZS equipped with a 4 mW laser operating at 633 nm. TEM and Cryo-EM measurements were performed on a Jeol JEM 1400 Transmission Electron Microscope with an operating voltage of 120 keV. No unexpected or unusually high safety hazards were encountered.

S2. Synthesis

S2.1 Synthesis of 4-(methylsulfinyl)phenol[1]

To 4-(methylthio)phenol (1.0 mmol) was added solution of 30% H_2O_2 (1.2 equiv., 0.04 g) and boric acid (10 mol%, 0.1 mmol, 0.006 g), and the mixture was stirred at room temperature for 30 min. The mixture was extracted with CH_2Cl_2 (5 × 10 mL) and the organic layers washed with brine (15 mL). The brine was extracted additional 5 times with CH_2Cl_2. The combined organics was dried over Na_2SO_4 and the solvent was removed through rotatory evaporation. The crude product was purified by flash chromatography over silica gel (methanol/ethyl acetate 2:98) and crystalized in ethyl acetate to obtain 4-(methylsulfinyl)phenol (62.0 mg, 0.40 mmol, 40% yield) as white crystals. ^1H NMR (400 MHz, $CDCl_3$): δ= 8.57 (s, 1H, OH), 7.51 (d, J = 8.5 Hz, 2H, Ar-H), 6.96 (d, J = 8.5 Hz, 2H, Ar-H), 2.76 (s, 3H, CH_3). ^{13}C NMR (101 MHz, $CDCl_3$): δ= 160.5 (C_q), 133.8 (C_q), 126.2 (CH, arom.), 116.9 (CH, arom.), 43.3 (CH_3). MS (ESI+) m/z: $[M + H]^+$ calcd. for $C_7H_8O_2S$, 157.03, found 156.93. The spectroscopic data are in accordance with those reported in literature.[2]

S2.2 Synthesis of 4-(methylthio)phenylacrylate

Triethylamine (Et_3N) (6.27 mL, 1.50 equiv.) was added dropwise to a solution of 4-(methylthio)phenol (4.20 g, 30.0 mmol, 1.00 equiv.) and acryloyl chloride (3.64 mL, 1.50 equiv.) in dry CH_2Cl_2 at 0 °C and stirred overnight for 16 hours, slowly increasing the temperature to 20 °C. The reaction mixture was

diluted with CH_2Cl_2 (250 mL) and washed with water (500 mL) and brine (500 mL). The combined organic layers were dried over anhydrous Na_2SO_4 and the solvent was removed under reduced pressure. The crude product was purified by flash chromatography over silica gel (ethyl acetate/petroleum ether 1:9 to 1:4) to afford 4-(methylthio)phenylacrylate (MTPA) (4.30 g, 22.1 mmol, 74% yield) as a light yellow oil. ^1H NMR (400 MHz, $CDCl_3$, δ): 7.29 (d, J = 8.4 Hz, 2H, Ar-H), 7.07 (d, J = 8.4 Hz, 2H, Ar-H), 6.60 (d, J = 17.3 Hz, 1H, $CHCH_2$), 6.31 (dd, J = 17.3, 10.4 Hz, 1H, CH_2), 6.01 (d, J = 10.4 Hz, 1H, CH_2), 2.49 (s, 3H, CH_3). ^{13}C NMR (101 MHz, $CDCl_3$, δ): 164.7 (C=O), 148.4 (C_q), 135.9 (C_q), 132.8 (CH, arom.), 128.2 (CH, arom.), 128.0 ($CHCH_2$), 122.1 (CH_2CH), 16.6 (CH_3). EIMS *m/z* (%):196 (1.5), 195 (3.7), 194 (32) [M^+], 142 (5.3), 141 (8.8), 140 (100) [$M^+ - C_3H_2O$], 139 (6), 125 (20), 96 (3.8), 55 (34), 45 (3.4). The spectroscopic data are in accordance with those reported in the literature.[3]

S2.3 Synthesis of p(DMA_n) macro-DDMAT

Specific conditions and data are shown in Table S1. N,N-dimethylacrylamide (DMA), which was filtered over basic alumina prior to use, and 2-(dodecylthiocarbonothioylthio)-2-methylpropionic acid (DDMAT) were dissolved in DMF and degassed for 15 minutes by bubbling argon gas. The resulting solution was stirred in a light reactor (444 nm), samples were taken regularly to follow the conversion with ^1H NMR. The reaction was stopped after 10 hours when the desired conversion was obtained. The reaction mixture was diluted with DMF (10.0 mL) and precipitated three times in diethyl ether (500 mL). The precipitated product was dried in a vacuum oven at 50 °C for three hours to afford p(DMA_{102}) macroDDMAT (2.46 g) and p(DMA_{130}) macroDDMAT (3.37 g) as yellow solids.

Table S1 pDMA_n synthesis and characterization data.

	[DDMAT]$_0$/ [DMA]$_0$	DMA (mmol)	DMA (g)	DDMAT (mmol)	DDMAT (g)	DMF (mL)	Reaction time (h)	^1H NMR Conversion (%)
**p(DMA_{102})**	1/120	40	4.0	0.35	0.12	5.9	10	85
**p(DMA_{130})**	1/200	70	6.9	0.35	0.12	6.8	10	65

S2.4 Synthesis of p(DMA_n-b-$MTPA_m$)

Specific conditions and data are shown in Table S2. Macro chain transfer agent p(DMA_n) macro-DDMAT and 4-(methylthio)phenyl acrylate were dissolved in DMF (1.0 mL) and degassed for 15 minutes by bubbling argon gas. The resulting solution was stirred in a light reactor (444 nm) for the given time. The reaction mixture was diluted with DMF (5.00 mL) and precipitated three times in diethyl ether (300 mL). The precipitated product was dried in a vacuum oven at 50 °C for three hours to afford p(DMA_{102}-b-$MTPA_{32}$) (0.54 g) and p(DMA_{130}-b-$MTPA_{16}$) (1.13 g) as white solids.

Table S2 p(DMA_n-b-$MTPA_m$) synthesis and characterization data.

	[p(DMA_n) macro-DDMAT]$_0$/ [MTPA]$_0$	p(DMA_n) macro-DDMAT (mmol)	p(DMA_n) macro-DDMAT (g)	MTPA (mmol)	MTPA (g)	DMF (mL)	Reaction time (h)	^1H NMR Conversi on (%)
**p(DMA_{102}) b-$MTPA_{32}$**	1/66	0.12	1.3	7.9	1.5	5.3	13	48
**p(DMA_{130}) b-$MTPA_{16}$**	1/25	0.10	1.3	2.4	0.46	6.8	6	64

S3. Polymer Characterization

For all the polymers, ^1H NMR spectra were recorded in $CDCl_3$, and the molecular weight was measured through gel permeation chromatography (GPC) in DMF. p(DMA_n-b-$MTPA_m$) structures were derived by ^1H NMR as reported below.

Polymerization conversion (ρ) was calculated by monitoring reduction in the ^1H NMR integrals of the monomer unsaturated protons (∫M: 5.60 – 6.80 ppm for DMA, 6.12 – 6.55 ppm for MTPA) and aromatic

protons in case of MTPA (7.32 ppm) relative to the proton (7.95 ppm) of the reaction solvent DMF. The ^1H NMR spectra to follow the polymerization conversion were taken in DMSO-d6. In the case of a copolymerization with both DMA and MTPA the conversion of both monomers was calculated according Equation S1.

$$\rho = \frac{\int M\,(t0) - \int M\,(t)}{\int M\,(t0)} \qquad \textbf{Equation S1}$$

For a polymerization containing z monomers, $M_{n,conv}$ was calculated according to Equation S2. Here $[Mx]_0$ is the initial concentration of monomer *x*, $[CTA]_0$ is the initial chain transfer agent (CTA) concentration and M_{Mx} and M_{CTA} are the monomer *x* and CTA molecular weights, respectively.

$$M_{n,conv} = \sum_{x=1}^{Z} \rho * \frac{[M]_0}{[CTA]_0} * M_{Mx} + M_{CTA} \qquad \textbf{Equation S2}$$

S3.1 ^1H NMR spectra of the block copolymers

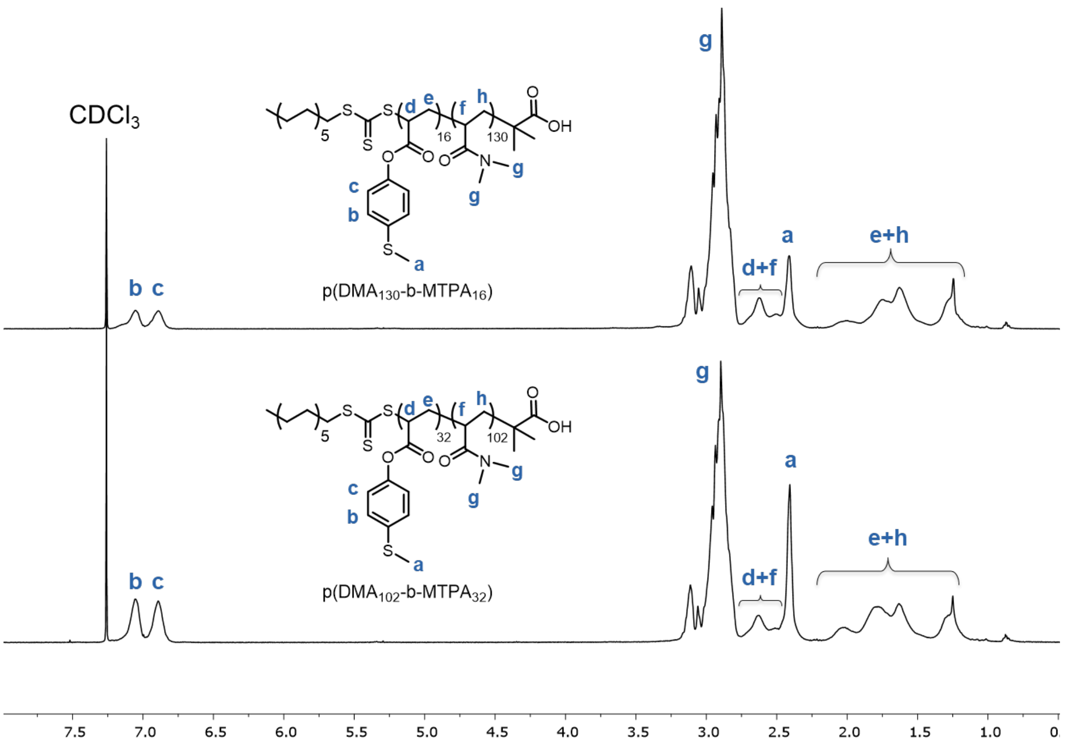

Figure S1. ^1H NMR stacked spectra of p(DMA_{130}-b-$MTPA_{16}$) (top) and poly(DMA_{102}-b-$MTPA_{32}$) (bottom) in $CDCl_3$.

S3.2 GPC data of the block copolymers

The average molecular weight and dispersity Đ (M_w/M_n) of the synthesized polymers was measured using a Shimadzu GPC with DMF LiBr (25 mM) as eluent. The system was equipped with a Shimadzu CTO-20AC Column oven, a Shimadzu RID-10A refractive index detector, a Shimadzu SPD-20A UV-Vis detector, PL gel guard column (MIXED, 5 µm), 50 mm x 7.5 mm, and 1× Agilent PLGel (MIXED, 5 µm), 300 mm × 7.5 mm, providing an effective molar mass range of 200 to 2 x 10^6 g/mol. DMF LiBr (25 mM) was used as an eluent with a flow rate of 1.0 mL/min at 50 °C. The GPC columns were calibrated with low dispersity PMMA standards (Sigma Aldrich) ranging from 800 to 2.2 x 10^6 g/mol, and molar masses are reported as PMMA equivalents. A 3rd-order polynomial was used to fit the log Mp vs. time calibration curve for both systems, which was near linear across the molar mass ranges.

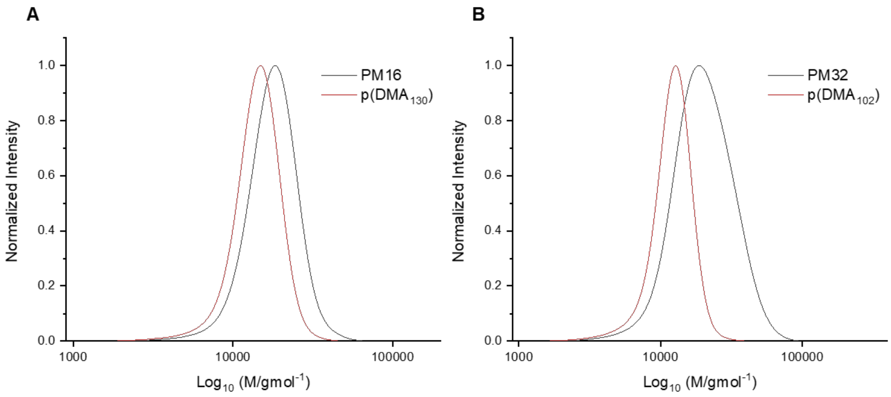

Figure S2. A) GPC traces of p(DMA_{130}) and the chain extended p(DMA_{130}-b-$MTPA_{16}$). B) GPC traces of p(DMA_{102}) and the chain extended p(DMA_{102}-b-$MTPA_{32}$).

S4. Characterization of polymeric micelles

S4.1 Preparation of the polymeric micelles

10 mg of p(DMA_m-b-$MTPA_n$) was dissolved in THF (0.25 mL), and sodium phosphate buffer (PB, 10 mL, 100 mM, pH = 7.4) was added slowly while vigorously stirring. The suspended micelles were left to stir for 18 hours in an open vial to evaporate the organic solvent.

S4.2 DLS measurements of the polymeric micelles before and after H_2O_2 treatment

To 1.0 mL of a 1.0 mg/mL micellar dispersion of p(DMA_m-b-$MTPA_n$) prepared as previously described were added 66 µL of stock solutions of hydrogen peroxide in PB with variable concentration to yield a final H_2O_2 concentration of 0.0, 0.007, 0.2, 2.0 wt%. The size distribution and the scattering intensity at 37°C were followed by DLS as a function of time. The curves are drawn for the different data sets as a guide for the eye.

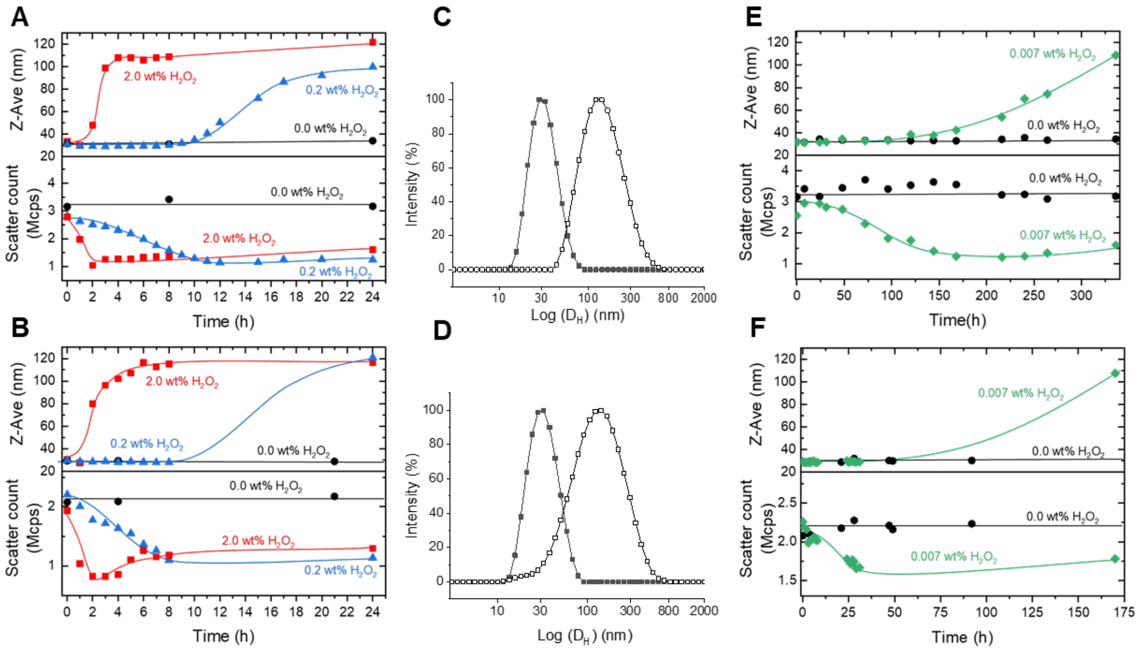

Figure S3. PM16 micelles in presence of different H_2O_2 concentrations at 37 °C. The curves are drawn as a guide for the eye. A) Z-Average size (top) and scatter count (bottom) distributions of PM16 micelles measured by DLS for three concentrations of H_2O_2: 2.0 wt% (■), 0.2 wt% (▲) and 0.0 wt% (control ●). B) Z-Average size (top) and scatter count (bottom) distributions of PM16 micelles (repeated with independent batch) measured by DLS for three concentrations of H_2O_2: 2.0 wt% (■), 0.2wt% (▲) and 0.0 wt% (control ●). C) Intensity plot measured by DLS of PM16 micelles before (■) and 24 h after (□) the addition of 2.0 wt% H_2O_2. D) Intensity plot measured by DLS of PM16 micelles before (■) and 24 h after (□) the addition of 0.2 wt% H_2O_2. E) Z-Average size (top) and scatter count (bottom) distributions of PM16 micelles measured by DLS after addition of 0.007 wt% (2 mM) H_2O_2 (♦) compared with control (●) over 336 h. F) Z-Average size (top) and scatter count (bottom) distributions of PM16 micelles (repeated with independent batch) measured by DLS after addition of 0.007 wt% (2 mM) H_2O_2 (♦) compared with control (●) over 170 h.

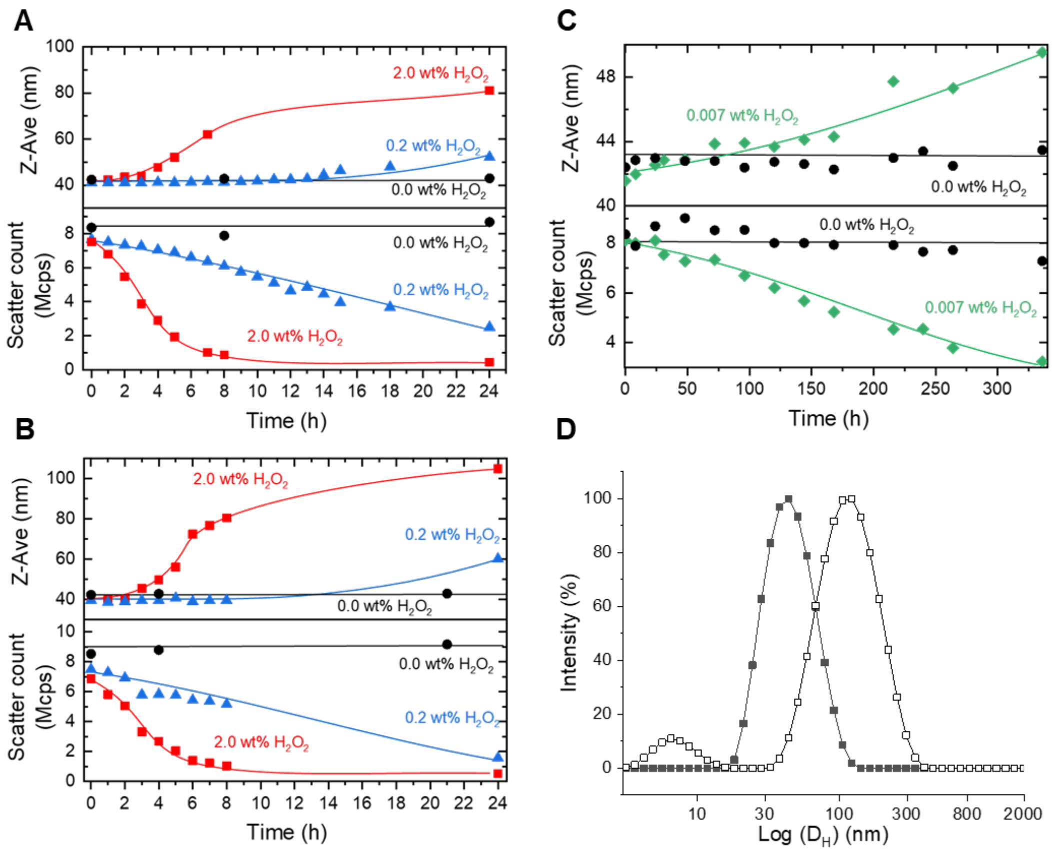

Figure S4. PM32 micelles in presence of different H_2O_2 concentrations at 37 °C. The curves are drawn as a guide for the eye. A) Z-Average size (top) and scatter count (bottom) distributions of PM32 micelles measured by DLS for three concentrations of H_2O_2: 2.0 wt% (■), 0.2 wt% (▲) and 0.0 wt% (control ●). B) Z-Average size (top) and scatter count (bottom) distributions of PM32 micelles (repeated with independent batch) measured by DLS for three concentrations of H_2O_2: 2.0 wt% (■), 0.2 wt% (▲) and 0.0 wt% (control ●). C) Z-Average size (top) and scatter count (bottom) distributions of PM32 micelles measured by DLS after addition of 0.007 wt% (2 mM) H_2O_2 (♦) compared with control (●) over 336 h. D) Intensity plot measured by DLS of PM32 micelles before (■) and 24 h after (□) the addition at of 2.0 wt% H_2O_2.

S4.3 TEM images of the polymeric micelles before and after H_2O_2 treatment

To 1.0 mL of a 1.0 mg/mL micellar dispersion of p(DMA_m-b-$MTPA_n$) prepared as previously described was added 66 μL of stock solutions of H_2O_2 in phosphate buffer (100 mM, pH = 7.4) with variable concentration to yield a final H_2O_2 concentration of 0.0 and 2.0 wt%. After 24 hours the samples were prepared for TEM by adding 3.0 μL p(DMA_m-b-$MTPA_n$) micelles solution onto a Formvar/Carbon 400 mesh Cu grid. 3.0 μL uranyl acetate stain (2 wt% in H_2O) was pipetted on the grid, which was then washed with Milli-Q water and dried on filter paper 3 times. The grid was finally loaded on the TEM single tilt holder to acquire the pictures of the samples. For the statistical analysis of the micelles diameters, about 20 images were made of each of the samples. TEM images were analysed manually using ImageJ.[4]

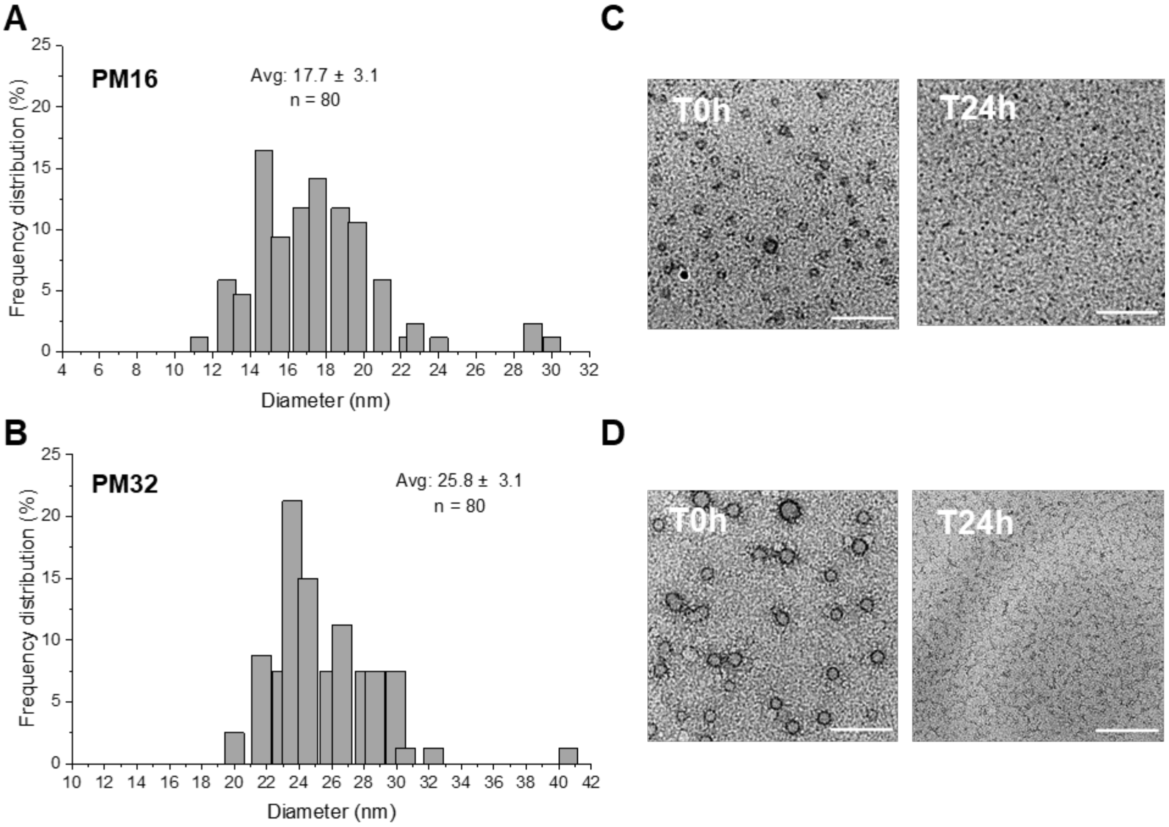

Figure S5 TEM images and particle analysis of PM16 and PM32 micelles stained with 2.0 wt% uranyl acetate. A) Normalized frequency distribution based on TEM images analysis of PM16 micelles. B) Normalized frequency distribution based on TEM images analysis of PM32 micelles. C) TEM images (Scale bar = 100 nm) of PM16 micelles before (left) and 24 h after (right) the addition of 2.0 wt% H_2O_2. D) TEM images (Scale bar = 100 nm) of PM32 micelles before (left) and 24 h after (right) the addition of 2.0 wt% H_2O_2.

S4.4 Cryo-EM images of the polymeric micelles before and after H_2O_2 treatment

To two vials was added 2.0 mL each of a 1.0 mg/mL micellar dispersion of p(DMA_m-b-$MTPA_n$) prepared as previously described. To one of these vials, 132 µL of a stock solution of H_2O_2 in PB (100 mM, pH = 7.4) was added to reach final H_2O_2 concentration of 0.2 wt% to take Cryo-EM pictures after 24 hours. To the second vial, 132 µL of phosphate buffer (100 mM, pH = 7.4) was added to obtain the sample without H_2O_2. The samples were centrifuged (4000 rpm for 15 minutes) using 10 kDa filters and concentrated to 20 mg/mL afterwards. The concentrated in 100 µL volume was washed with additional 100 µL H_2O, obtaining a final concentration of 10 mg/mL.

Cryo-TEM images were obtained by adding 4 µL of the 10 mg/mL micellar solution onto a Quantifoil 1.2/1.3 200 mesh Cu grid. The drop was blotted for four seconds with filter paper to obtain a thin layer on the grid, and vitrified by rapid immersion in liquid ethane (Leica EM GP version 16222032). The grid was finally inserted into a cryo-holder (Gatan model 626) and then transferred to the Jeol JEM 1400 TEM. For the statistical analysis of the micelles diameters, about 20 images were made of each of the samples. Cryo-EM images were analyzed manually using ImageJ.

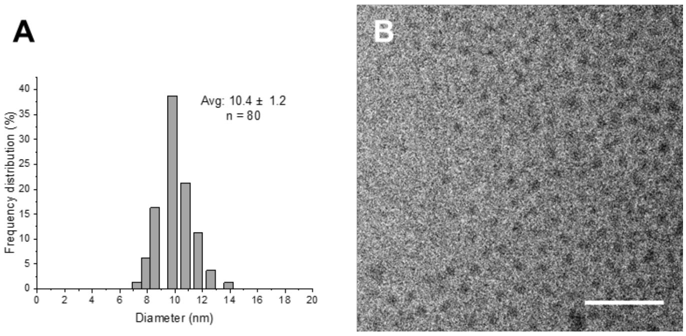

Figure S6 Cryo-EM images and particle analysis of PM16 micelles A) Normalized frequency distribution based on Cryo-EM images analysis of PM16 micelles. B) Cryo-EM image (Scale bar = 100 nm) of PM16 micelles.

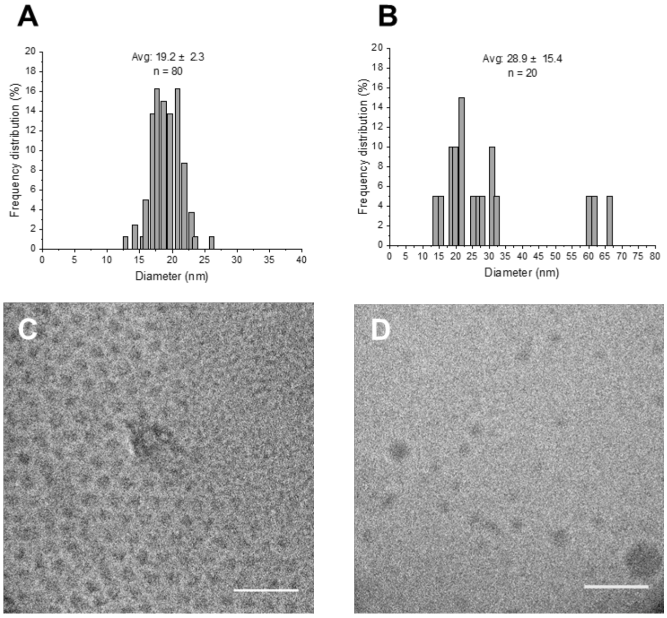

Figure S7 Cryo-EM images and particle analysis of PM32 micelles before and 24 h after the addition of 0.2 wt% H_2O_2. A) Normalized frequency distribution based on Cryo-EM images analysis of PM32 micelles. B) Normalized frequency distribution based on Cryo-EM images analysis of PM32 micelles 24 h after the addition of 0.2 wt% H_2O_2. C) Cryo-EM image (Scale bar = 100 nm) of PM32 micelles. D) Cryo-EM image (Scale bar = 100 nm) of PM32 micelles 24 h after the addition of 0.2 wt% H_2O_2.

S5. ^{1}H NMR study of p(DMA$_n$-b-MTPA$_m$) micelles

S5.1 ^{1}H NMR study of p(DMA$_n$-b-MTPA$_m$) micelles before and after H_2O_2 treatment

To 0.5 ml of p(DMA$_m$-b-MTPA$_n$) micelles (8.0 mg/mL) in a NMR tube was added 55 µL D_2O and 33 µL H_2O_2 (30 wt%). NMR tubes were kept at 37 °C during all the experiments. The first ^{1}H NMR spectrum was taken right after the addition of H_2O_2 (t=0) and, subsequently, a measurement is taken every hour until no change in conversion was detected. The conversion (%) of **1** and **2** was measured calculating the percentage of the integral of the respective aromatic peaks at 7.64 and 7.77 ppm for each time point against the total integral value obtained at the end of the acquisitions. The peak between 2.87 and 3.24 ppm corresponding to the protons of p(DMA$_n$) was used as reference.

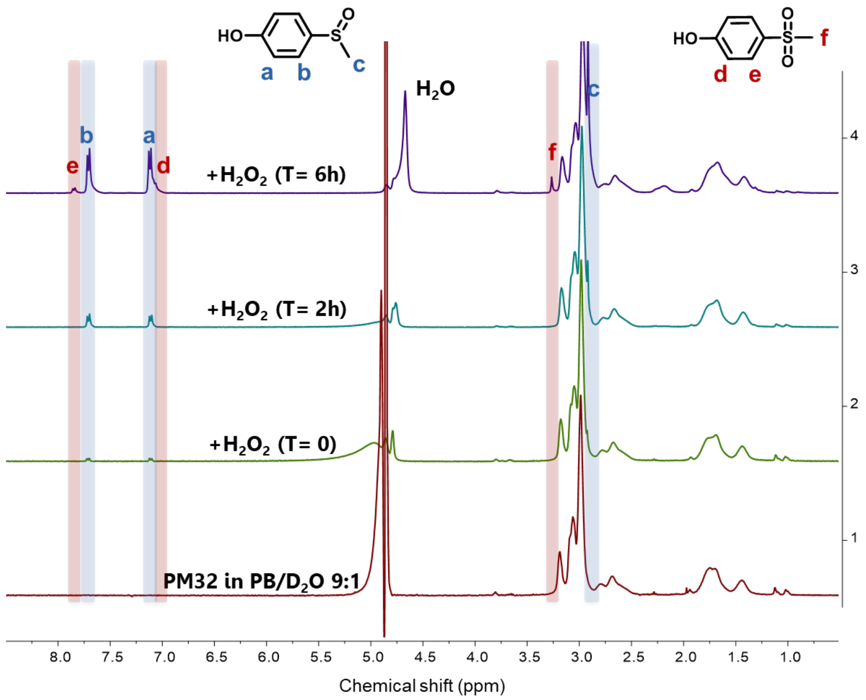

Figure S8. ^{1}H NMR of PM32 micelles after treatment with 2.0 wt% of H_2O_2 in PB (100mM, pH = 7.4) at 37 °C.

S5.2 ^{1}H NMR study of p(DMA$_n$-b-MTPA$_m$) micelles at different pH

p(DMA$_m$-b-MTPA$_n$) micelles (8.0 mg/mL) were prepared separately in PB (100 mM) with different pH: 5.0, 6.0, 7.4. From each micellar dispersion, 0.5 mL were added in a NMR tube together with 55 µL D_2O and 33 µL of the appropriate phosphate buffer at 37 °C. ^{1}H NMR measurements were taken every 24 h for 6 days. The conversion (%) of **1** was measured calculating the percentage of the difference between the integral of the peak in the aromatic region at 7.64 ppm for each time point and the integral of the same region at t=0, divided by the expected integral for the complete removal of **1**. The peak between 2.87 and 3.24 ppm corresponding to the protons of p(DMA$_n$) was used as reference.

S6. Drug load and release of p(DMA$_n$-b-MTPA$_m$) micelles

S6.1 Determination of micelle loading with Nile Red

A Nile Red solution in THF (20 µL, 1.0 mg/mL) was added to the previously prepared micellar dispersions of p(DMA$_m$-b-MTPA$_n$) (1.0 mL, 1.0 mg/mL) and incubated in the dark in an open vial to evaporate the organic solvent. The nonencapsulated payload was removed through centrifugation (5000 rpm, 10 minutes) and 900 µL DMF were added to 100 µL of the Nile Red-loaded micellar dispersions. The fluorescence of the solution was measured at an excitation wavelength of 540 ± 20 nm and emission wavelength 620 ± 30 nm and compared to the calibration curve of known concentrations Nile Red in PB (100 mM, pH = 7.4)/ DMF 1:9 (Figure S9), to determine the Nile Red loading per mg of polymer. Drug loading (DL) and encapsulation efficiency (EE) were calculated as follows (Equations S3 and S4, respectively).

$$\mathrm{DL(w/w)} = \frac{\text{amount of loaded drug}}{\text{amount of polymer}} \qquad \textbf{Equation S3}$$

$$\mathrm{EE(w/w\%)} = \frac{\text{actual amount of loaded drug}}{\text{theoretical amount of loaded drug}} \qquad \textbf{Equation S4}$$

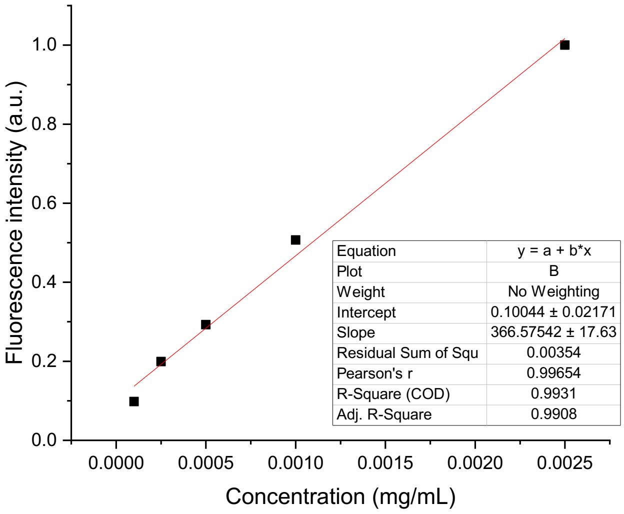

Figure S9. Nile Red calibration curve in phosphate buffer (100 mM, pH = 7.4)/ DMF 1:9.

Table S3. Properties of PM16 and PM32 micellar dispersions (1.0 mg/mL). The average hydrodynamic diameter (DH) is determined with DLS. The drug loading (DL) and encapsulation efficiency (EE) of Nile Red in the micelles were determined with fluorescence spectroscopy.

Polymer	D_H [unloaded] (nm)	DL (µg/mg polymer)	EE (%)
PM16	31.6 ± 0.5	2.4	12
PM32	42.4 ± 0.9	3.6	18

S6.2 H_2O_2-triggered release of Nile Red from p(DMA$_n$-b-MTPA$_m$) micelles

A Nile Red solution in THF (20 µL, 1.0 mg/mL) was added to the previously prepared micellar dispersions (1.0 mL, 1.0 mg/mL) and incubated in the dark in an open vial to evaporate the organic solvent. The micellar dispersions were divided over three vials for each polymer and hydrogen peroxide solution was added to a final concentration of 0, 0.2 and 2.0 wt% for each series. The fluorescence of the solution was measured on a Synergy H1 (Biotek) microplate reader at 37 °C, using an excitation wavelength of 540 ± 20 nm and emission wavelength 620 ± 30 nm. The release percentage for each sample at specific time point was determined by subtracting the fluorescent value (F_t) from that of the sample before the addition of H_2O_2 (F_{t0}), and the percent fluorescence remaining was determined by normalization to the same value (F_{t0}).

S7. Cell viability assay on p(DMA$_n$-b-MTPA$_m$) micelles

HeLa cells in DMEM culture medium supplemented with 10% fetal bovine serum (Gibco, life technologies^{TM}) and 1% Penicillin/Streptomycin (100x, Biowest) under humidified normoxic (95% air, 5% CO_2) were plated at 2000 cells/well (suspended in 200 µL cell culture medium) in a 96-well plate and incubated at 37 °C. After 3 days, 20 µL of both PM16 and PM32 micelles (0.0-11 mg/mL) in PBS (phosphate buffer saline, pH = 7.4) was added to each well, to reach final micelles concentrations in the range (0.0-1000 µg /mL). After 24 hours, the micellar solutions were removed, the cells were washed with PBS for three times, and 200 µL of fresh culture medium was added. The cells have been allowed to grow for an additional 3 days, then their cytotoxicity was evaluated using the WST-8 assay (Cell Counting Kit-8, Dojindo Laboratories, Tebu-Bio). For this test, 10 µL of CCK-8 reagent was added to each well and incubated for 3 hours, then the absorbance at 450 nm was measured using a microplate scanning spectrophotometer (PowerWave XSTM, Bio-Tek). The surviving fraction (SF) of the Hela Cells was calculated according equation S5.

$$\mathrm{SF} = \frac{Abs(450)_{sample} - Abs(450)_{blank}}{Abs(450)_{control} - Abs(450)_{blank}}$$ ....**Equation S5**

Abs(450)sample is the absorbance at 450 nm for cell incubated with p(DMA_n-b-$MTPA_m$) micelles;
Abs(450)control is the absorbance at 450 nm for cell incubated with 10 µL of PBS;
Abs(450) blank is the absorbance at 450 nm for vials without addition of CCK-8 reagent.

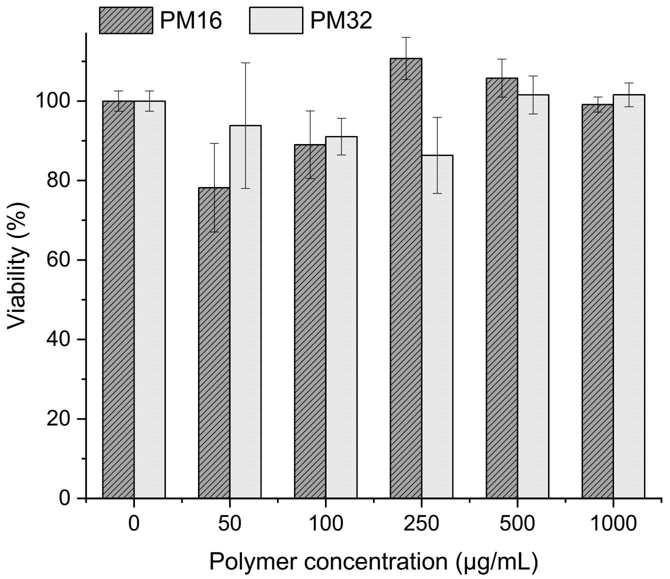

Figure S10. PM16 and PM32 cytocompatibility in HeLa cells cultured in DMEM culture medium. Cells were treated with micellar dispersions between 0.0 and 1.0 mg/mL in phosphate buffered saline (PBS) (20 uL/220 uL well). After 24 h cell viability was measured by WST-8 assay.

References

1 A. Rostami and J. Akradi, *Tetrahedron Lett.*, 2010, **51**, 3501.

2 F. Secci, A. Frongia and P. P. Piras, *Tetrahedron Lett.*, 2014, **55**, 603.

3 R. Kakuchi and P. Theato, *Macromolecules*, 2012, **45**, 1331.

4 C. A. Schneider, W. S. Rasband and K. W. Eliceiri, *Nat. Methods*, 2012, **9**, 671.

S8.0 ^{1}H-NMR and ^{13}C-NMR spectrum for synthesized compounds.

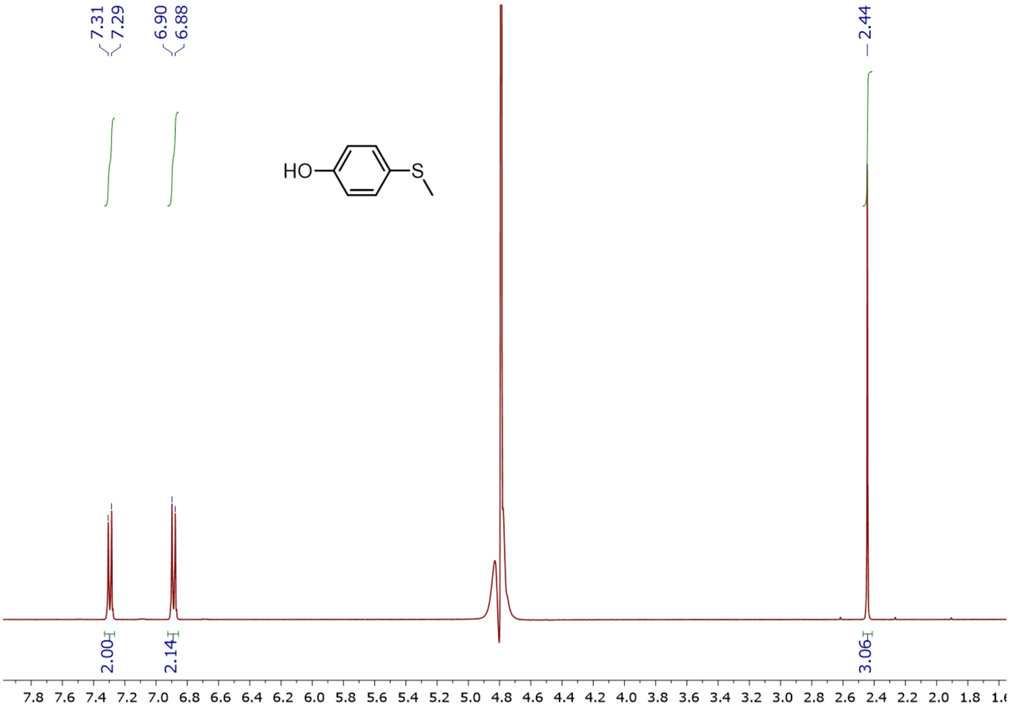

^{1}H NMR spectrum (400 MHz) of 4-(methylthio)phenol in PB (100 mM, pH=7.4)/D_2O 9:1.

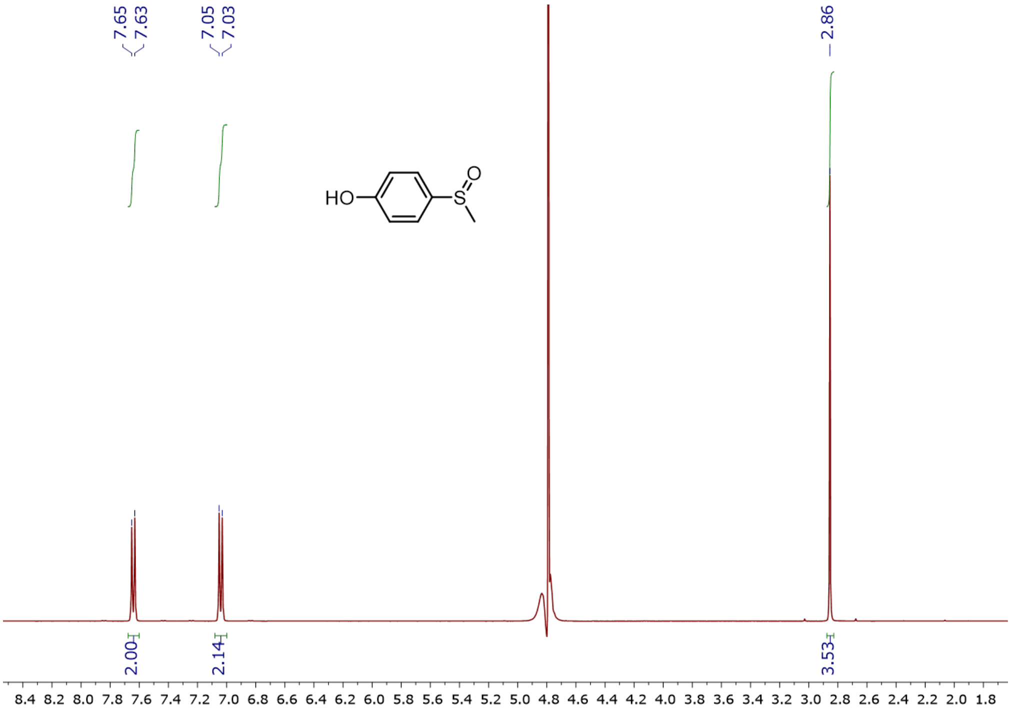

^{1}H NMR spectrum (400 MHz) of 4-(methylsulfinyl)phenol in PB (100 mM, pH=7.4)/D_2O 9:1.

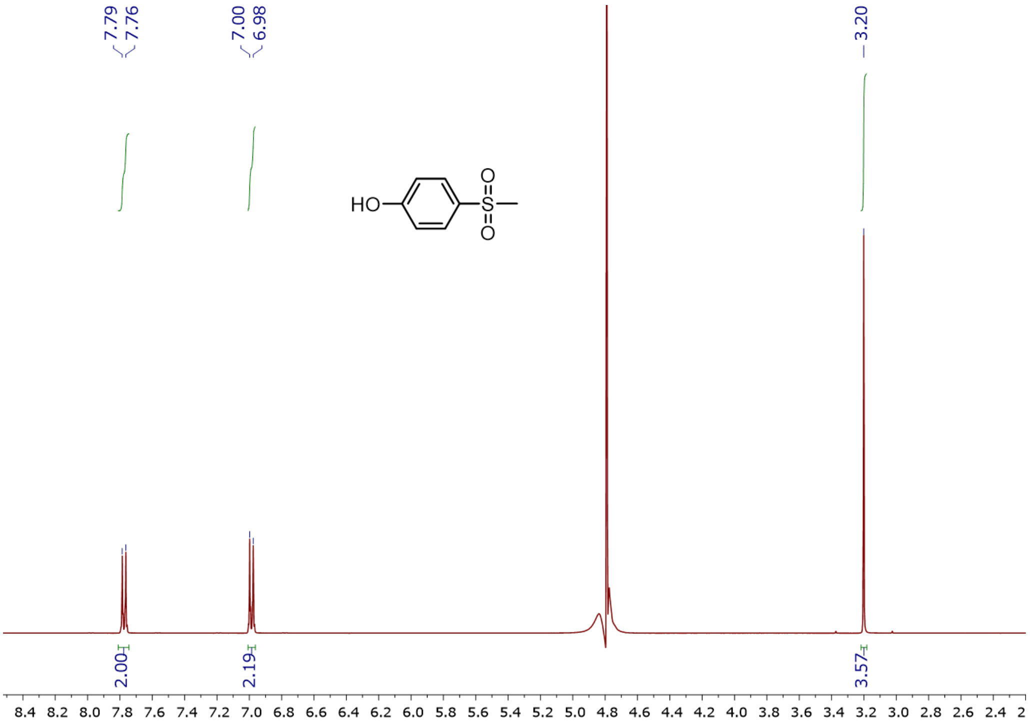

^{1}H NMR spectrum (400 MHz) of 4-(methylsulfonyl)phenol in PB (100 mM, pH=7.4)/D_2O 9:1.

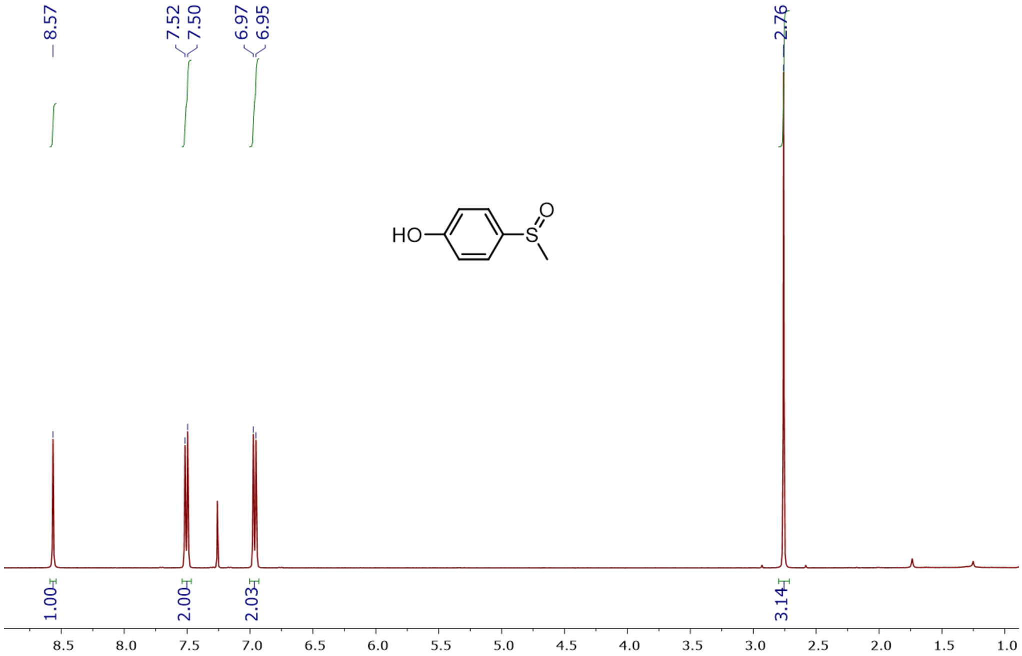

^{1}H NMR spectrum (400 MHz) of 4-(methylsulfinyl)phenol in $CDCl_3$.

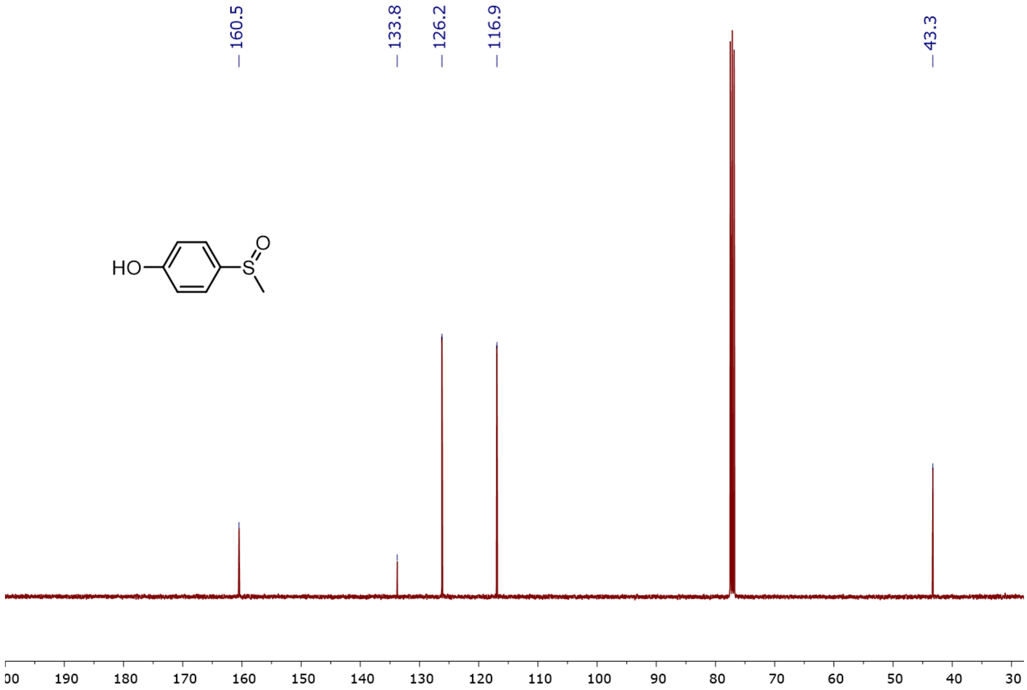

^{13}C NMR spectrum (101 MHz) of 4-(methylsulfinyl)phenol in $CDCl_3$.

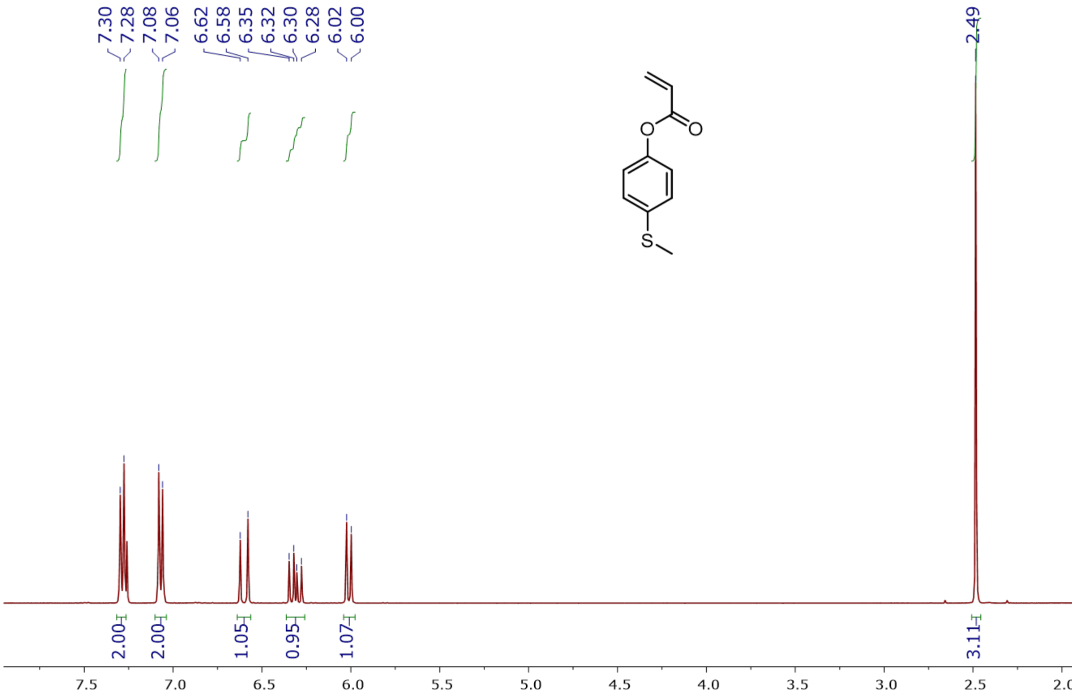

^{1}H NMR spectrum (400 MHz) of 4-(methylthio)phenyl acrylate in $CDCl_3$.

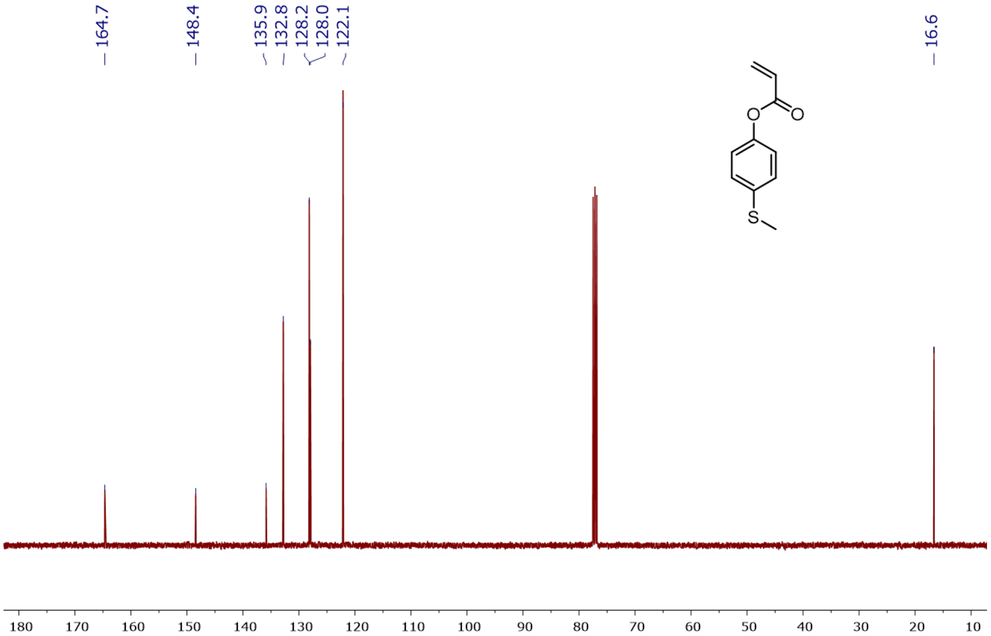

^{13}C NMR spectrum (101 MHz) of 4-(methylthio)phenyl acrylate in $CDCl_3$.

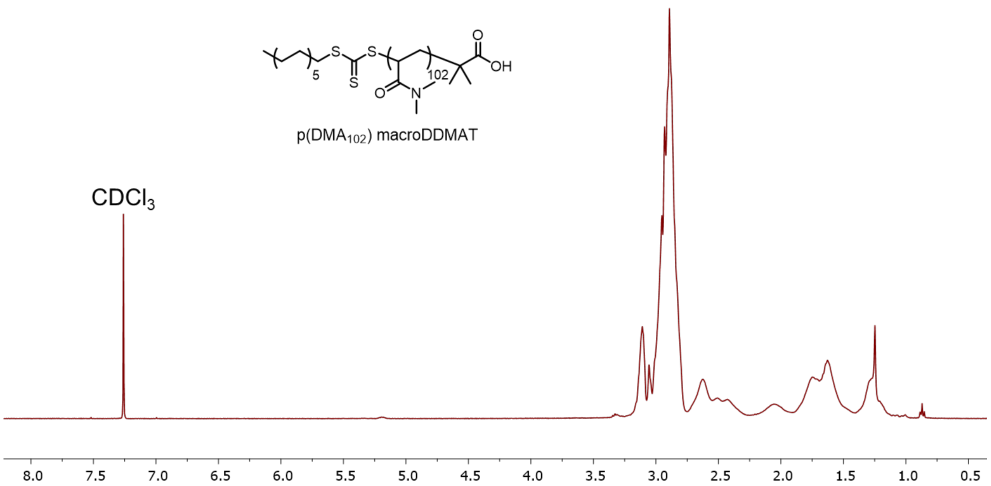

^{1}H NMR spectrum (400 MHz) of p(DMA_{102}) macroDDMAT in $CDCl_3$.

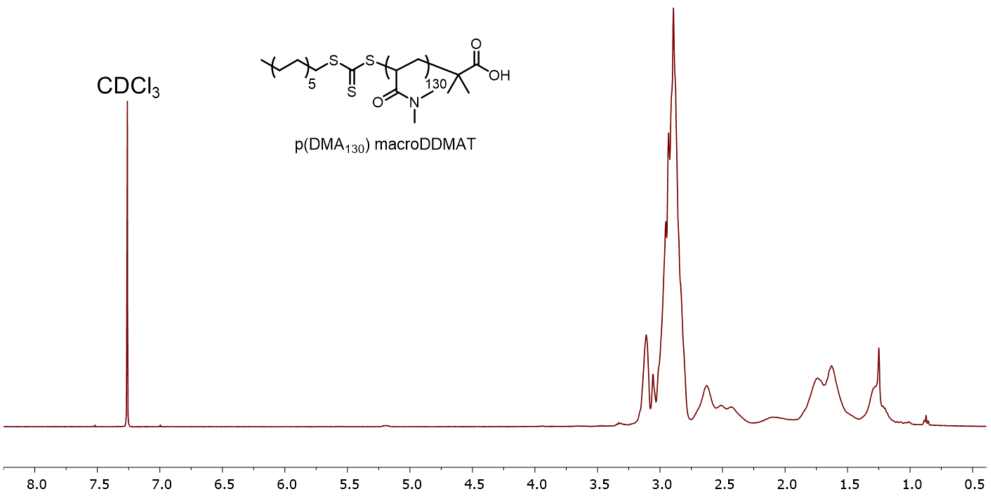

^{1}H NMR spectrum (400 MHz) of p(DMA_{130}) macroDDMAT in $CDCl_3$.
